# On a Matrix Inequality Related to the Distillability Problem

**DOI:** 10.3390/e20080588

**Published:** 2018-08-08

**Authors:** Yi Shen, Lin Chen

**Affiliations:** 1 School of Mathematics and Systems Science, Beihang University, Beijing 100191, China; 2 International Research Institute for Multidisciplinary Science, Beihang University, Beijing 100191, China

**Keywords:** matrix inequality, singular value, eigenvalue, Kronecker product, quantum information, distillability problem, 15A18, 15A21, 15A45, 15A69

## Abstract

We investigate the distillability problem in quantum information in ${\mathbb{C}}^{d}⊗{\mathbb{C}}^{d}$. One case of the problem has been reduced to proving a matrix inequality when d=4. We investigate the inequality for three families of non-normal matrices. We prove the inequality for the first two families with d=4 and for the third family with d≥5. We also present a sufficient condition for the fulfillment of the inequality with d=4.

## 1. Introduction

Extracting pure entanglement from mixed states is a basic task in quantum information. This task is called entanglement distillation formally. We first briefly introduce the physical motivation of the distillability problem. In quantum physics, a quantum state is mathematically described by a positive semidefinite matrix. The state is pure when it has rank one, otherwise the state is mixed. Pure entangled states play an essential role in most quantum-information tasks such as quantum computation. Nevertheless, there is no pure state in nature due to the inevitable decoherence between the state and environment. Therefore, asymptotically converting initially bipartite entangled mixed states into bipartite pure entangled states under local operations and classical communications (LOCC) is a key step in quantum information processing. The distillability problem [1,2] asks whether the above-mentioned conversion succeeds for any mixed states. It has been a main open problem in quantum information [1] for a long time, since the distillability problem lies at the heart of entanglement theory [3,4,5] and is related to the separability problem extensively studied by the quantum information community recently [6,7,8]. There have been some attempts at the problem in the past years [1,2,9,10,11,12,13,14,15,16].

In the following, we will show a mathematical description of the distillability problem. Let $ℋ={ℋ}_{A}⊗{ℋ}_{B}$ be the bipartite Hilbert space with $\mathrm{Dim}{ℋ}_{A}=M$ and $\mathrm{Dim}{ℋ}_{B}=N$. Recall that a quantum state is a positive semidefinite matrix. For the sake of normalization in quantum physics, it is required that every quantum state be a unit vector. However, the requirement does not play an essential role in the distillability problem and often causes inconvenience in the mathematical expressions and discussion. In this paper, unless stated otherwise, the states will not be normalized.

We shall work with the quantum state ρ on *ℋ*. Such a ρ is called a bipartite state of system *A* and *B*. We have $\rho =\sum_{i,j=1}^{M}E_{ij}⊗\rho_{ij}$, where $E_{ij}$ is an M×M matrix, the elements of which are all zero, except that the (i,j) entry is one. The partial transpose of ρ with respect to the system *A* is defined as $\rho^{\Gamma }:=\sum_{i,j=1}^{M}E_{ji}⊗\rho_{ij}$. We say that ρ is the positive partial transpose (PPT) if $\rho^{\Gamma }\geq 0$. Otherwise, ρ is the negative partial transpose (NPT), i.e., $\rho^{\Gamma }$ has at least one negative eigenvalue. The NPT states are entangled states due to the Peres–Horodecki criterion in quantum information [17,18]. We say that a quantum state is pure when it has rank one.

Since the distillability problem requires many copies of the same states, we further need the concept of a composite system. Let $\rho_{A_{i}B_{i}}$ be an $M_{i}\times N_{i}$ state of rank $r_{i}$ acting on the Hilbert space ${ℋ}_{A_{i}}⊗{ℋ}_{B_{i}}$, i=1,2, with $\mathrm{Dim}{ℋ}_{A_{i}}=M_{i}$ and $\mathrm{Dim}{ℋ}_{B_{i}}=N_{i}$. Suppose ρ of systems $A_{1},A_{2}$ and $B_{1},B_{2}$ is a state on the Hilbert space ${ℋ}_{A_{1}}⊗{ℋ}_{B_{1}}⊗{ℋ}_{A_{2}}⊗{ℋ}_{B_{2}}$, such that the partial trace ${\mathrm{Tr}}_{A_{1}B_{1}}\rho =\rho_{A_{2}B_{2}}$ and ${\mathrm{Tr}}_{A_{2}B_{2}}\rho =\rho_{A_{1}B_{1}}$. By switching the two middle factors, we can regard ρ as a composite bipartite state on the Hilbert space ${ℋ}_{A}⊗{ℋ}_{B}$ where ${ℋ}_{A}={ℋ}_{A_{1}}⊗{ℋ}_{A_{2}}$ and ${ℋ}_{B}={ℋ}_{B_{1}}⊗{ℋ}_{B_{2}}$. We write $\rho =\rho_{A_{1}A_{2}:B_{1}B_{2}}$. One can verify that ρ is an $M_{1}M_{2}\times N_{1}N_{2}$ state of rank at most $r_{1}r_{2}$. For example, the tensor product $\rho =\rho_{A_{1}B_{1}}⊗\rho_{A_{2}B_{2}}$ is an $M_{1}M_{2}\times N_{1}N_{2}$ state of rank $r_{1}r_{2}$. The above definition can be generalized to the tensor product of *N* states $\rho_{A_{i}B_{i}},i=1,\ldots ,N$. They form a bipartite state on the Hilbert space ${ℋ}_{A_{1},\cdots ,A_{N}}⊗{ℋ}_{B_{1},\cdots ,B_{N}}$. It is written as ${ℋ}^{⊗n}$ with ${ℋ}_{A_{i}}⊗{ℋ}_{B_{i}}=ℋ$.

Third, we shall refer to the notations ⏐*ψ*〉 and 〈*ψ*⏐ in quantum physics respectively as a column vector and its conjugate transpose in linear algebra. In quantum information, the well-known Werner state in $C^{d}⊗C^{d}$ is defined as $\frac{I+\alpha \sum_{i,j=1}^{d}E_{ij}⊗E_{ji}}{d^{2}+\alpha d}$, where the real number α∈[−1,1] [19]. We introduce the definition of distillable states as follows [1].

**Definition** **1.** *A bipartite state ρ is n-distillable under local operations and classical communications if there exists a Schmidt-rank-two bipartite state *⏐*ψ*〉*$\in {ℋ}^{⊗n}$ such that *〈*ψ*⏐*${\left(\rho^{⊗n}\right)}^{\Gamma }|\psi \rangle <0$. Otherwise, we say that ρ is n-undistillable. We say that ρ is distillable if it is n-distillable for some n≥1.*

The definition shows that PPT states are not distillable. It has been shown [1] that all NPT bipartite states can be locally converted into NPT Werner states. Using Definition 1, one can show that the distillability of NPT Werner states is equivalent to that with α=−1/2. Therefore, the distillability problem indeed asks whether Werner states with α=−1/2 are distillable. In this paper, we investigate one case of the distillability problem, i.e., the two-undistillability of Werner states in $C^{4}⊗C^{4}$ with α=−1/2. It is known that this case is equivalent to the following mathematical problem [9].

Let $A\in C^{n\times n}$ and $B\in C^{m\times m}$. Denote *A*^†^ and *B*^†^ as the conjugate transpose of *A* and *B*, respectively. The Kronecker sum of *A* and *B* denoted by $A{⊕}_{K}B$ is defined as $A⊗I_{m}+I_{n}⊗B$; see more facts in [20] (Section 7.2). The work in [9] has presented the following conjecture on the Kronecker sum when *A* and *B* have the same size.

**Conjecture** **1.** *Let*$A,B,I\in C^{d\times d}$, d≥4*and the matrix:*

(1) $$\begin{array}{c} X=A⊗I+I⊗B \end{array}$$

*where:*

(2) $$\begin{array}{c} \mathrm{Tr}A=\mathrm{Tr}B=0,\;\mathrm{Tr}A^{†}A+\mathrm{Tr}B^{†}B=\frac{1}{d}. \end{array}$$

*Define set* $X_{d}$ *whose elements are given by Equations (1) and (2). Let* $\sigma_{1},\cdots ,\sigma_{d^{2}}$ *be the singular values of* $X\in X_{d}$ *in the descending order. Then:*

(3) $$\begin{array}{c} \underset{X\in X_{d}}{\sup}\;\left(\sigma_{1}^{2}+\sigma_{2}^{2}\right)\leq \frac{1}{2}. \end{array}$$

The condition d≥4 is essential as one can show that Conjecture 1 fails for d=3 from Lemma A1. It has been shown [9] that Conjecture 1 for d=4 is the mathematical description of a two-copy distillability problem. They are equivalent through a given state-operator isomorphism ([9], Equation (43)). It is also known that Conjecture 1 holds for all normal matrices with d≥4 [9]. Evidently, the matrix *X* in (1) is normal if and only if *A* and *B* in (1) are both normal. The remaining work on Conjecture 1 is to prove it when *X* is non-normal. It turns out to be a hard problem and there is no progress so far, as far as we know.

In this paper, we investigate Conjecture 1 in terms of three families of non-normal matrices *X*. They are respectively constructed in Definitions 2–4. We prove Conjecture 1 for the first family $P_{1}$ of non-normal *X* in Theorem 1, based on Propositions 1 and 2, and for the second family $P_{2}$ of non-normal *X* in Theorem 2. For the third family $P_{3}$ of non-normal *X*, we prove Conjecture 1 with d≥5 in Theorem 3. We also present a sufficient condition for Conjecture 1 with d=4 in Lemma 10. It is an idea different from the above three families of matrices to deal with Conjecture 1. Our results carry out the first step to prove Conjecture 1 for non-normal matrices and, thus, the distillability problem in quantum information.

The rest of this paper is organized as follows. We introduce some notations and preliminary results in linear algebra in Section 2. We investigate Conjecture 1 for three families of non-normal matrices in Section 3.1, Section 3.2 and Section 3.3, respectively. We also show a sufficient condition for Conjecture 1 with d=4 in Section 3.4. Finally, we provide some discussion on the distillability problem and conclude our work in Section 4.

## 2. Preliminaries

We first present some mathematical notations. We shall denote $A^{*}$, $A^{T}$ and $A^{†}$ as the conjugate, transpose and conjugate transpose of matrix *A*, respectively. We refer to $C^{n\times n}$ as the set of n×n matrices with entries in the complex field and $H^{n\times n}$ as the set of n×n Hermitian matrices. Let $I_{n}$ be the identity matrix in $C^{n\times n}$. We shall omit the subscript of the identity matrix when it is clear in the paper. Let σ(A) be the spectrum of matrix *A*, $\lambda_{i}\left(A\right)$ be an eigenvalue of *A* and $A_{\left(i,j\right)}$ be the (i,j) entry of *A*. We shall say that *X* is unitarily similar to *Y*, i.e., X∼Y when there exists a unitary matrix *W* such that $X=WYW^{†}$. In particular, they are locally similar when there exist unitary matrices $U,V\in C^{d\times d}$ such that W=U⊗V. We denote this as $X{∼}_{L}Y$.

We then post some lemmas, which will be used in the following section. The following lemma is essential to simplify Conjecture 1.

**Lemma** **1.** *The following four statements are equivalent.*

*(i)*  *Conjecture 1 holds.*
*(ii)*  *Conjecture 1 holds when X is replaced by $X^{T}$, $X^{*}$ or X*^†^.
*(iii)*  *Conjecture 1 holds when X is replaced by any matrix unitarily similar to X.*
*(iv)*  *Conjecture 1 holds when X is replaced by I⊗A+B⊗I.*

**Proof** **of** **Lemma** **1.** The equivalences between (i) and (ii) and between (i) and (iii) follow from straightforward computation. We next prove that (i) is equivalent to (iv). It follows from a known fact of the Kronecker sum, i.e., for $A\in C^{n\times n}$ and $B\in C^{m\times m}$, there exists a permutation $P\in C^{mn\times mn}$ such that $P\left(A{⊕}_{K}B\right)P^{-1}=B{⊕}_{K}A$. Hence, $A{⊕}_{K}B$ and $B{⊕}_{K}A$ are unitarily similar when m=n, and thus, (i) is equivalent to (iv) by (iii). ☐

Using statement (iii), we can generally assume that *A* and *B* in Conjecture 1 are both upper-triangular. In particular, we can assume that they are diagonal if and only if they are normal.

Then, let us recall the result on normal matrices in [9]. We will briefly show its proof and some related new findings in Appendix A.

**Lemma** **2.** *Let $N_{d}$ be a subset of normal operators X in (1) satisfying the constraints (2). Then, Conjecture 1 holds for $X\in N_{d}$ where d≥4.*

The following lemmas are useful to prove the result of the third non-normal family.

**Lemma** **3** ([20], Fact 4.10.16.)**.** *(Gershgorin circle theorem) Let $A\in C^{n\times n}$. Then,*

(4) σ(A)⊂G(A)=⋃i=1ns∈C:|s−A(i,i)|≤∑j=1j≠in|A(i,j)|,

*and a corollary is:*

(5) $$\begin{array}{c} \sigma \left(A\right)\subset \overset{n}{\underset{i=1}{⋃}}\left\{s\in C:|s|\leq \sum_{j=1}^{n}|A_{\left(i,j\right)}|\right\}. \end{array}$$

**Remark** **1.** *Let Ri=∑j=1j≠in|A(i,j)| and $D(A_{\left(i,i\right)},R_{i})$ be the closed disc centered at $A_{\left(i,i\right)}$ with radius $R_{i}$. Every eigenvalue of A lies within at least one of the Gershgorin discs $D(A_{\left(i,i\right)},R_{i})$.*

**Lemma** **4** ([20], Fact 4.10.21.)**.** *(Brauer theorem) Let $A\in C^{n\times n}$. Then,*

(6) σ(A)⊂⋃i,j=1i≠jns∈C:|s−A(i,i)||s−A(j,j)≤∑k=1k≠in|A(i,k)|∑k=1k≠jn|A(j,k)|.

**Remark** **2.** *The eigenvalues of A lie in the union of n(n−1)/2 ovals of Cassini, which is contained in the union of Gershgorin discs (4). Hence, the Brauer theorem is stronger than the Gershgorin circle theorem.*

**Lemma** **5** ([21], Corollary 4.3.15.)**.** *Let $A,B\in H^{n\times n}$. Let the eigenvalues of A,B be in the increasing order, that is $\lambda_{1}\leq \lambda_{2}\leq \cdots \leq \lambda_{n}$. Then, for all i=1,⋯n, we have:*

(7) $$\begin{array}{c} \lambda_{i}\left(A\right)+\lambda_{1}\left(B\right)\leq \lambda_{i}\left(A+B\right)\leq \lambda_{i}\left(A\right)+\lambda_{n}\left(B\right). \end{array}$$

## 3. Results

In this section, we will present our main results on non-normal matrices. We prove Conjecture 1 with d=4 for the first two families we construct and prove Conjecture 1 with d≥5 for the third family we construct. We also show a sufficient condition for Conjecture 1 with d=4.

### 3.1. Conjecture 1 with Non-Normal Matrices X: Family 1

In this subsection, we prove Conjecture 1 with a family of non-normal matrices *X* in Definition 2. We will formulate our main result in Theorem 1, followed by two preliminary facts, i.e., Proposition 1 and 2.

**Definition** **2.** *Let $P_{1}$ be the subset of matrices X with d=4, such that A is normal or $A∼\left[\begin{array}{cccc} 0 & a_{1} & 0 & 0\\ a_{2} & 0 & 0 & 0\\ 0 & 0 & 0 & a_{3}\\ 0 & 0 & a_{4} & 0 \end{array}\right]$, and B is normal or $B∼\left[\begin{array}{cccc} 0 & b_{1} & 0 & 0\\ b_{2} & 0 & 0 & 0\\ 0 & 0 & 0 & b_{3}\\ 0 & 0 & b_{4} & 0 \end{array}\right]$. A and B should satisfy the constraints in (2).*

One can show $N_{4}\subset P_{1}$, and $\left[\begin{array}{cccc} 0 & a_{1} & 0 & 0\\ a_{2} & 0 & 0 & 0\\ 0 & 0 & 0 & a_{3}\\ 0 & 0 & a_{4} & 0 \end{array}\right]∼\left[\begin{array}{cccc} \tilde{a_{11}} & \tilde{a_{12}} & 0 & 0\\ 0 & \tilde{a_{22}} & 0 & 0\\ 0 & 0 & \tilde{a_{33}} & \tilde{a_{34}}\\ 0 & 0 & 0 & \tilde{a_{44}} \end{array}\right]$ with $\tilde{a_{11}}+\tilde{a_{22}}=\tilde{a_{33}}+\tilde{a_{44}}=0$. We present the main result of this section as follows.

**Theorem** **1.** *The inequality (3) holds when $X\in P_{1}$.*

**Proof** **of** **Theorem** **1.** If $A∼\left[\begin{array}{cccc} 0 & a_{1} & 0 & 0\\ a_{2} & 0 & 0 & 0\\ 0 & 0 & 0 & a_{3}\\ 0 & 0 & a_{4} & 0 \end{array}\right]$ and $B∼\left[\begin{array}{cccc} 0 & b_{1} & 0 & 0\\ b_{2} & 0 & 0 & 0\\ 0 & 0 & 0 & b_{3}\\ 0 & 0 & b_{4} & 0 \end{array}\right]$, then the assertion follows from Proposition 1. If one of *A* and *B* is normal, then we may assume that it is diagonal by Lemma 1 (iii). Therefore, the assertion follows from Proposition 2 and the switch of A,B (if any), as well, because of Lemma 1 (iv). If *A* and *B* are both normal, then the assertion follows from Lemma 2. This completes the proof.  ☐

**Proposition** **1.** *The inequality (3) holds when $X\in P_{1}$, $A∼\left[\begin{array}{cccc} 0 & a_{1} & 0 & 0\\ a_{2} & 0 & 0 & 0\\ 0 & 0 & 0 & a_{3}\\ 0 & 0 & a_{4} & 0 \end{array}\right]$ and $B∼\left[\begin{array}{cccc} 0 & b_{1} & 0 & 0\\ b_{2} & 0 & 0 & 0\\ 0 & 0 & 0 & b_{3}\\ 0 & 0 & b_{4} & 0 \end{array}\right]$.*

**Proof** **of** **Proposition** **1.** For Lemma 1 (iii), we assume $A=\left[\begin{array}{cccc} 0 & a_{1} & 0 & 0\\ a_{2} & 0 & 0 & 0\\ 0 & 0 & 0 & a_{3}\\ 0 & 0 & a_{4} & 0 \end{array}\right]$ and $B=\left[\begin{array}{cccc} 0 & b_{1} & 0 & 0\\ b_{2} & 0 & 0 & 0\\ 0 & 0 & 0 & b_{3}\\ 0 & 0 & b_{4} & 0 \end{array}\right]$. By computing, one can show that $X^{†}X=Y_{1}⊕Y_{2}$. We can partition $Y_{1}$ as $\left[\begin{array}{cc} Z_{1} & Z_{2}\\ Z_{2}^{†} & Z_{4} \end{array}\right]$, where $Z_{1}=\mathrm{diag}\left(|{a_{2}|}^{2}+{\left|b_{2}\right|}^{2},{\left|a_{2}\right|}^{2}+{\left|b_{1}\right|}^{2},{\left|a_{2}\right|}^{2}+{\left|b_{4}\right|}^{2},{\left|a_{2}\right|}^{2}+{\left|b_{3}\right|}^{2}\right)$, $Z_{2}=\left[\begin{array}{cccc} 0 & a_{1}b_{2}^{*}+b_{1}a_{2}^{*} & 0 & 0\\ a_{1}b_{1}^{*}+b_{2}a_{2}^{*} & 0 & 0 & 0\\ 0 & 0 & 0 & a_{1}b_{4}^{*}+b_{3}a_{2}^{*}\\ 0 & 0 & a_{1}b_{3}^{*}+b_{4}a_{2}^{*} & 0 \end{array}\right]$, and $Z_{4}=\mathrm{diag}\left({\left|a_{1}\right|}^{2}+{\left|b_{2}\right|}^{2},{\left|a_{1}\right|}^{2}+{\left|b_{1}\right|}^{2},{\left|a_{1}\right|}^{2}+{\left|b_{4}\right|}^{2},{\left|a_{1}\right|}^{2}+{\left|b_{3}\right|}^{2}\right)$. One can formulate the characteristic polynomial of $Y_{1}$ as follows.

(8) $$\det\left(\lambda I-Y_{1}\right)=f_{1}\left(\lambda \right)\cdot f_{2}\left(\lambda \right)\cdot f_{3}\left(\lambda \right)\cdot f_{4}\left(\lambda \right),$$

where:

(9) $$\begin{array}{cc} f_{1}\left(\lambda \right) & =\left(\lambda -(|a_{1}{|}^{2}+|b_{2}{|}^{2})\right)\left(\lambda -(|a_{2}{|}^{2}+|b_{1}{|}^{2})\right)-{\left|a_{1}b_{1}^{*}+a_{2}^{*}b_{2}\right|}^{2},\\ f_{2}\left(\lambda \right) & =\left(\lambda -(|a_{1}{|}^{2}+|b_{1}{|}^{2})\right)\left(\lambda -(|a_{2}{|}^{2}+|b_{2}{|}^{2})\right)-{\left|a_{1}b_{2}^{*}+a_{2}^{*}b_{1}\right|}^{2},\\ f_{3}\left(\lambda \right) & =\left(\lambda -(|a_{1}{|}^{2}+|b_{4}{|}^{2})\right)\left(\lambda -(|a_{2}{|}^{2}+|b_{3}{|}^{2})\right)-{\left|a_{1}b_{3}^{*}+a_{2}^{*}b_{4}\right|}^{2},\\ f_{4}\left(\lambda \right) & =\left(\lambda -(|a_{1}{|}^{2}+|b_{3}{|}^{2})\right)\left(\lambda -(|a_{2}{|}^{2}+|b_{4}{|}^{2})\right)-{\left|a_{1}b_{4}^{*}+a_{2}^{*}b_{3}\right|}^{2}. \end{array}$$

We first claim the larger root of $f_{i}\left(\lambda \right)=0,\forall i=1,2,3,4$, is not greater than $\frac{1}{4}$. Take $f_{1}\left(\lambda \right)$ as an example. From the Vieta theorem, we have the sum of two roots of $f_{1}\left(\lambda \right)=0$ is $|{a_{1}|}^{2}+{\left|a_{2}\right|}^{2}+{\left|b_{1}\right|}^{2}+{\left|b_{2}\right|}^{2}$. Since $X^{†}X=Y_{1}⊕Y_{2}$ is a semipositive definite matrix, all the eigenvalues of $Y_{1}$ and $Y_{2}$ are non-negative. This implies that all the roots of $f_{i}\left(\lambda \right)=0,i=1,2,3,4$ are non-negative. So we have the larger root of $f_{1}\left(\lambda \right)=0$ is not greater than the sum of two roots, i.e., ${\left|a_{1}\right|}^{2}+{\left|a_{2}\right|}^{2}+{\left|b_{1}\right|}^{2}+{\left|b_{2}\right|}^{2}$. Recall that $\sum_{i=1}^{4}\left({\left|a_{i}\right|}^{2}+{\left|b_{i}\right|}^{2}\right)=\frac{1}{4}$. Therefore, we conclude that the larger root of $f_{1}\left(\lambda \right)=0$ is not greater than $\frac{1}{4}$. One can draw the same conclusion for $f_{2}\left(\lambda \right),f_{3}\left(\lambda \right),f_{4}\left(\lambda \right)$. Then, our claim holds. This implies the largest eigenvalue of $Y_{1}$ is no greater than $\frac{1}{4}$. One can show that $Y_{2}$ can be evolved from $Y_{1}$ by replacing $a_{1}$ with $a_{3}$ and replacing $a_{2}$ with $a_{4}$ in $Y_{1}$. Hence, we obtain the expression of $\det(\lambda I-Y_{2})$ by replacing $a_{1}$ with $a_{3}$ and replacing $a_{2}$ with $a_{4}$ in (9). In the same way, we conclude that the largest eigenvalue of $Y_{2}$ is no greater than $\frac{1}{4}$. Since $X^{†}X=Y_{1}⊕Y_{2}$, the sum of the largest two eigenvalues of $X^{†}X$ is at most $\frac{1}{2}$. This completes the proof. ☐

We proceed with the proof of the upcoming Proposition 2. For this purpose, we need two preliminary results. The first result is known as one of the basic inequalities.

**Lemma** **6.** *If a,b,x,y∈R then $ab{\left(x+y\right)}^{2}\leq \left(a+b\right)\left(ax^{2}+by^{2}\right)$.*

**Lemma** **7.** *Suppose $a_{1},a_{2},b_{1},b_{2}$ are nonnegative real numbers and $a_{1}^{2}+a_{2}^{2}+b_{1}^{2}+b_{2}^{2}=1/4$. Then:*

(10) $$\sqrt{{\left(a_{1}^{2}-a_{2}^{2}\right)}^{2}+4b_{1}^{2}{\left(a_{1}+a_{2}\right)}^{2}}+\sqrt{{\left(a_{1}^{2}-a_{2}^{2}\right)}^{2}+4b_{2}^{2}{\left(a_{1}+a_{2}\right)}^{2}}\leq 1/2.$$

**Proof** **of** **Lemma** **7.** Using the basic inequality $x+y\leq \sqrt{2(x^{2}+y^{2})}$ for any real x,y, we obtain that the lhs of (10) is upper bounded by:

(11) $$\begin{array}{cc} & \sqrt{2\left(2{\left(a_{1}^{2}-a_{2}^{2}\right)}^{2}+4\left(b_{1}^{2}+b_{2}^{2}\right){\left(a_{1}+a_{2}\right)}^{2}\right)}\\ = & \sqrt{2{\left(a_{1}+a_{2}\right)}^{2}\left(1-2{\left(a_{1}+a_{2}\right)}^{2}\right)}\\ \leq & 1/2. \end{array}$$

The equality follows from the equation $a_{1}^{2}+a_{2}^{2}+b_{1}^{2}+b_{2}^{2}=1/4$. This completes the proof. ☐

**Proposition** **2.** *The inequality (3) holds when $X\in P_{1}$, for which $A∼\left[\begin{array}{cccc} 0 & a_{1} & 0 & 0\\ a_{2} & 0 & 0 & 0\\ 0 & 0 & 0 & a_{3}\\ 0 & 0 & a_{4} & 0 \end{array}\right]$ and B is normal, i.e., $B∼\mathrm{diag}(b_{1},b_{2},b_{3},b_{4})$.*

**Proof** **of** **Proposition** **2.** For Lemma 1 (iii), we assume $A=\left[\begin{array}{cccc} 0 & a_{1} & 0 & 0\\ a_{2} & 0 & 0 & 0\\ 0 & 0 & 0 & a_{3}\\ 0 & 0 & a_{4} & 0 \end{array}\right]$ and $B=\mathrm{diag}(b_{1},b_{2},b_{3},b_{4})$. Since *A* and *B* satisfy (2), we have:

(12) $$\begin{array}{ccc} \sum_{i=1}^{4}b_{i} & = & 0, \end{array}$$

(13) $$\begin{array}{ccc} \sum_{j=1}^{4}\left({\left|a_{j}\right|}^{2}+{\left|b_{j}\right|}^{2}\right) & = & 1/4. \end{array}$$

By computing, one can show that $X^{†}X=H_{1}⊕H_{2}$. We can partition $H_{1}$ and $H_{2}$ as follows.

(14) $$H_{1}=\left[\begin{array}{cc} D_{11} & D_{12}\\ D_{12}^{†} & D_{14} \end{array}\right],H_{2}=\left[\begin{array}{cc} D_{21} & D_{22}\\ D_{22}^{†} & D_{24} \end{array}\right],$$

where:

(15) $$\begin{array}{cc} D_{11} & \mathrm{diag}=(|a_{2}{|}^{2}+|b_{1}{|}^{2},|a_{2}{|}^{2}+|b_{2}{|}^{2},|a_{2}{|}^{2}+|b_{3}{|}^{2},|a_{2}{|}^{2}+|b_{4}{|}^{2}),\\ D_{12} & \mathrm{diag}=(a_{1}b_{1}^{*}+a_{2}^{*}b_{1},a_{1}b_{2}^{*}+a_{2}^{*}b_{2},a_{1}b_{3}^{*}+a_{2}^{*}b_{3},a_{1}b_{4}^{*}+a_{2}^{*}b_{4}),\\ D_{14} & \mathrm{diag}=(|a_{1}{|}^{2}+|b_{1}{|}^{2},|a_{1}{|}^{2}+|b_{2}{|}^{2},|a_{1}{|}^{2}+|b_{3}{|}^{2},|a_{1}{|}^{2}+|b_{4}{|}^{2}),\\ D_{21} & \mathrm{diag}=(|a_{4}{|}^{2}+|b_{1}{|}^{2},|a_{4}{|}^{2}+|b_{2}{|}^{2},|a_{4}{|}^{2}+|b_{3}{|}^{2},|a_{4}{|}^{2}+|b_{4}{|}^{2}),\\ D_{22} & \mathrm{diag}=(a_{3}b_{1}^{*}+a_{4}^{*}b_{1},a_{3}b_{2}^{*}+a_{4}^{*}b_{2},a_{3}b_{3}^{*}+a_{4}^{*}b_{3},a_{3}b_{4}^{*}+a_{4}^{*}b_{4}),\\ D_{24} & \mathrm{diag}=(|a_{3}{|}^{2}+|b_{1}{|}^{2},|a_{3}{|}^{2}+|b_{2}{|}^{2},|a_{3}{|}^{2}+|b_{3}{|}^{2},|a_{3}{|}^{2}+|b_{4}{|}^{2}). \end{array}$$

There exists a permutation $P=\left[\begin{array}{cccccccc} 1 & 0 & 0 & 0 & 0 & 0 & 0 & 0\\ 0 & 0 & 0 & 1 & 0 & 0 & 0 & 0\\ 0 & 0 & 0 & 0 & 0 & 1 & 0 & 0\\ 0 & 0 & 0 & 0 & 0 & 0 & 1 & 0\\ 0 & 1 & 0 & 0 & 0 & 0 & 0 & 0\\ 0 & 0 & 1 & 0 & 0 & 0 & 0 & 0\\ 0 & 0 & 0 & 0 & 1 & 0 & 0 & 0\\ 0 & 0 & 0 & 0 & 0 & 0 & 0 & 1 \end{array}\right]⊕\left[\begin{array}{cccccccc} 1 & 0 & 0 & 0 & 0 & 0 & 0 & 0\\ 0 & 0 & 0 & 1 & 0 & 0 & 0 & 0\\ 0 & 0 & 0 & 0 & 0 & 1 & 0 & 0\\ 0 & 0 & 0 & 0 & 0 & 0 & 1 & 0\\ 0 & 1 & 0 & 0 & 0 & 0 & 0 & 0\\ 0 & 0 & 1 & 0 & 0 & 0 & 0 & 0\\ 0 & 0 & 0 & 0 & 1 & 0 & 0 & 0\\ 0 & 0 & 0 & 0 & 0 & 0 & 0 & 1 \end{array}\right]$ such that $P^{†}(X^{†}X)P={⊕}_{j=1}^{4}\left(Y_{j}⊕Z_{j}\right)$, where $Y_{j}$ and $Z_{j}$ are order-two submatrices such that:

(16) $$Y_{j}=\left[\begin{array}{cc} |a_{2}{|}^{2}+{\left|b_{j}\right|}^{2} & b_{j}^{*}a_{1}+a_{2}^{*}b_{j}\\ b_{j}a_{1}^{*}+a_{2}b_{j}^{*} & |a_{1}{|}^{2}+{\left|b_{j}\right|}^{2} \end{array}\right],$$

and:

(17) $$Z_{j}=\left[\begin{array}{cc} |a_{4}{|}^{2}+{\left|b_{j}\right|}^{2} & b_{j}^{*}a_{3}+a_{4}^{*}b_{j}\\ b_{j}a_{3}^{*}+a_{4}b_{j}^{*} & |a_{3}{|}^{2}+{\left|b_{j}\right|}^{2} \end{array}\right].$$

Let λ and μ be two arbitrary eigenvalues of $X^{†}X$. Then, proving the inequality (3) is equivalent to proving λ+μ≤1/2. There are five cases for λ and μ.

Case 1. λ and μ are the eigenvalues of the same $Y_{j}$ or $Z_{j}$. Equations (13) and (16) imply that $\lambda +\mu =\mathrm{Tr}Y_{j}\leq 1/2$. Equations (13) and (17) imply that $\lambda +\mu =\mathrm{Tr}Z_{j}\leq 1/2$.

Case 2. λ and μ are the eigenvalues of different $Y_{j}$’s. Without loss of generality, we can assume that λ is the maximum eigenvalue of $Y_{1}$ and μ is the maximum eigenvalue of $Y_{2}$. By computation, one can obtain:

(18) $$\begin{array}{ccc} \lambda & = & \frac{1}{2}\left(|a_{1}{|}^{2}+|a_{2}{|}^{2}+2{\left|b_{1}\right|}^{2}+\sqrt{\left(\right|a_{1}{|}^{2}-|a_{2}{|}^{2}{)}^{2}+4{\left|b_{1}a_{2}^{*}+a_{1}b_{1}^{*}\right|}^{2}}\right), \end{array}$$

(19) $$\begin{array}{ccc} \mu & = & \frac{1}{2}\left(|a_{1}{|}^{2}+|a_{2}{|}^{2}+2{\left|b_{2}\right|}^{2}+\sqrt{\left(\right|a_{1}{|}^{2}-|a_{2}{|}^{2}{)}^{2}+4{\left|b_{2}a_{2}^{*}+a_{1}b_{2}^{*}\right|}^{2}}\right). \end{array}$$

Therefore, λ+μ is upper bounded by the sum of the rhs of (18) and (19), in which any $a_{i}$ and $b_{j}$ are replaced by $|a_{i}|$ and $|b_{j}|$, respectively, and $a_{3},a_{4},b_{3},b_{4}$ equal zero. Using Lemma 7 and (13), we have λ+μ≤1/2.

Case 3. λ and μ are the eigenvalues of different $Z_{j}$’s. We can prove Conjecture 1 by following the proof in Case 2, except that we switch $a_{1}$ and $a_{3}$ and switch $a_{2}$ and $a_{4}$ at the same time.

Case 4. λ is the eigenvalue of some $Y_{j}$; μ is the eigenvalue of some $Z_{k}$; and j≠k. Without loss of generality, we may assume that j=1 and k=2. By computation, one can show that:

(20) $$\begin{array}{ccc} \lambda +\mu & = & \frac{1}{2}\left(\right|a_{1}{|}^{2}+|a_{2}{|}^{2}+|a_{3}{|}^{2}+|a_{4}{|}^{2}+2(|b_{1}{|}^{2}+|b_{2}{|}^{2})\\ & + & \sqrt{\left(\right|a_{1}{|}^{2}-|a_{2}{|}^{2}{)}^{2}+4{\left|b_{1}a_{2}^{*}+a_{1}b_{1}^{*}\right|}^{2}}+\sqrt{\left(\right|a_{3}{|}^{2}-|a_{4}{|}^{2}{)}^{2}+4{\left|b_{2}a_{4}^{*}+a_{3}b_{2}^{*}\right|}^{2}})\\ & \leq & \frac{1}{2}\left(\right|a_{1}{|}^{2}+|a_{2}{|}^{2}+|a_{3}{|}^{2}+|a_{4}{|}^{2}+2(|b_{1}{|}^{2}+|b_{2}{|}^{2})\\ & + & \sqrt{\left(\right|a_{1}{|}^{2}-|a_{2}{|}^{2}{)}^{2}+4|b_{1}{|}^{2}\left(\right|a_{1}\left|+\right|a_{2}{\left|\right)}^{2}}+\sqrt{\left(\right|a_{3}{|}^{2}-|a_{4}{|}^{2}{)}^{2}+4|b_{2}{|}^{2}\left(\right|a_{3}\left|+\right|a_{4}{\left|\right)}^{2}})\\ & \leq & \frac{1}{2}\left(\right|a_{1}{|}^{2}+|a_{2}{|}^{2}+|a_{3}{|}^{2}+|a_{4}{|}^{2}+2(|b_{1}{|}^{2}+|b_{2}{|}^{2})\\ & + & \sqrt{\left(\left(\right|a_{1}\left|+\right|a_{2}{\left|\right)}^{2}+\left(\right|a_{3}\left|+\right|a_{4}{\left|\right)}^{2}\right)\left(\left(\right|a_{1}\left|-\right|a_{2}{\left|\right)}^{2}+\left(\right|a_{3}\left|-\right|a_{4}{\left|\right)}^{2}+4(|b_{1}{|}^{2}+|b_{2}{|}^{2})\right)})\\ & := & \frac{1}{2}\left(x+\sqrt{y(2x-y)}\right)\\ & \leq & x\\ & \leq & 1/2, \end{array}$$

where $x=|a_{1}{|}^{2}+|a_{2}{|}^{2}+|a_{3}{|}^{2}+|a_{4}{|}^{2}+2(|b_{1}{|}^{2}+|b_{2}{|}^{2})$. The second inequality in (20) follows from Lemma 6 in which we have set $x=\sqrt{\left(\right|a_{1}{|}^{2}-|a_{2}{|}^{2}{)}^{2}+4|b_{1}{|}^{2}\left(\right|a_{1}\left|+\right|a_{2}{\left|\right)}^{2}}$, $y=\sqrt{\left(\right|a_{3}{|}^{2}-|a_{4}{|}^{2}{)}^{2}+4|b_{2}{|}^{2}\left(\right|a_{3}\left|+\right|a_{4}{\left|\right)}^{2}}$, $a={\left(a_{3}+a_{4}\right)}^{2}$ and $b={\left(a_{1}+a_{2}\right)}^{2}$. The last inequality in (20) follows from (13).

Case 5. λ is the eigenvalue of some $Y_{j}$, and μ is the eigenvalue of $Z_{j}$. Without loss of generality, we may assume that j=1. Equations (12) and (13) imply that $|b_{1}{|}^{2}=|b_{2}+b_{3}+b_{4}{|}^{2}\leq 3(|b_{2}{|}^{2}+|b_{3}{|}^{2}+|b_{4}{|}^{2})=\frac{3}{4}-3|b_{1}{|}^{2}-3\sum_{j=1}^{4}{\left|a_{j}\right|}^{2}.$ Hence:

(21) $$|b_{1}{|}^{2}\leq \frac{3}{16}-\frac{3}{4}\sum_{j=1}^{4}{\left|a_{j}\right|}^{2}.$$

On the other hand, by computation, one can show that:

(22) $$\begin{array}{ccc} \lambda +\mu & = & \frac{1}{2}\left(\right|a_{1}{|}^{2}+|a_{2}{|}^{2}+|a_{3}{|}^{2}+|a_{4}{|}^{2}+4{\left|b_{1}\right|}^{2}\\ & + & \sqrt{\left(\right|a_{1}{|}^{2}-|a_{2}{|}^{2}{)}^{2}+4{\left|b_{1}a_{2}^{*}+a_{1}b_{1}^{*}\right|}^{2}}+\sqrt{\left(\right|a_{3}{|}^{2}-|a_{4}{|}^{2}{)}^{2}+4{\left|b_{1}a_{4}^{*}+a_{3}b_{1}^{*}\right|}^{2}})\\ & \leq & \frac{1}{2}\left(\right|a_{1}{|}^{2}+|a_{2}{|}^{2}+|a_{3}{|}^{2}+|a_{4}{|}^{2}+4{\left|b_{1}\right|}^{2}\\ & + & \sqrt{\left(\right|a_{1}{|}^{2}-|a_{2}{|}^{2}{)}^{2}+4|b_{1}{|}^{2}\left(\right|a_{1}\left|+\right|a_{2}{\left|\right)}^{2}}+\sqrt{\left(\right|a_{3}{|}^{2}-|a_{4}{|}^{2}{)}^{2}+4|b_{1}{|}^{2}\left(\right|a_{3}\left|+\right|a_{4}{\left|\right)}^{2}})\\ & := & \Lambda (|a_{1}|,|a_{2}|,|a_{3}|,|a_{4}|,|b_{1}{|}^{2}). \end{array}$$

It monotonically increases with $|b_{1}{|}^{2}$. Using (13), we may assume that $|a_{1}|=x\cos d\cos g$, $|a_{2}|=x\cos d\sin g$, $|a_{3}|=x\sin d\cos h$ and $|a_{4}|=x\sin d\sin h$, where the real numbers x∈[0,1/2], and d,g,h∈[0,π/2]. Equations (21) and (22) imply that:

(23) $$\begin{array}{ccc} \lambda +\mu & \leq & \Lambda (|a_{1}|,|a_{2}|,|a_{3}|,|a_{4}|,\frac{3}{16}-\frac{3}{4}\sum_{j=1}^{4}|a_{j}{|}^{2})\\ & = & \frac{1}{8}\left(3-8x^{2}+2x\cos d\sqrt{f_{1}\left(d,x,g\right)}+2x\sin d\sqrt{f_{2}\left(d,x,h\right)}\right) \end{array}$$

where:

(24) $$\begin{array}{c} f_{1}\left(d,x,g\right)=3-10x^{2}+2x^{2}\cos2d+\left(3-12x^{2}\right)\sin2g+\left(-2x^{2}-2x^{2}\cos2d\right)\sin^{2}2g, \end{array}$$

and:

(25) $$\begin{array}{c} f_{2}\left(d,x,h\right)=3-10x^{2}-2x^{2}\cos2d+\left(3-12x^{2}\right)\sin2h+\left(-2x^{2}+2x^{2}\cos2d\right)\sin^{2}2h. \end{array}$$

One can verify that $f_{1}\left(d,x,g\right)=f_{2}\left(/2-d,x,g\right)$. The last equation of (23) is unchanged under the switch of *d* and π/2−d and the switch of *g* and *h* at the same time. Therefore, the maximum of (23) is achieved when x∈[0,1/2], d∈[0,π/4] and g,h∈[0,π/2].

To prove the assertion, one needs to obtain the maximum of (23). For this purpose, we need to obtain the maximum of the function $f_{1}$ in terms of *g* and the maximum of the function $f_{2}$ in terms of *h*. The two functions $f_{1}$ and $f_{2}$ are quadratic functions in terms of sin2g and sin2h, respectively. The axises of symmetry of $f_{1}$ and $f_{2}$ are respectively $\sin2g=\frac{3-12x^{2}}{4x^{2}+4x^{2}\cos2d}$ and $\sin2h=\frac{3-12x^{2}}{4x^{2}-4x^{2}\cos2d}$. If sin2g=1 or sin2h=1, then we respectively obtain $x=\frac{1}{\sqrt{4+\frac{8}{3}\cos^{2}d}}$ or $x=\frac{1}{\sqrt{4+\frac{8}{3}\sin^{2}d}}$. We discuss three subcases in terms of the above facts, sin2g≤1, sin2h≤1 and cosd≥sind.

Subcase 5.1. $x\in [0,\frac{1}{\sqrt{4+\frac{8}{3}\cos^{2}d}}]$. One can show that $\max_{g}f_{1}\left(d,x,g\right)=f_{1}\left(d,x,\pi /4\right)$ and $\max_{h}f_{2}\left(d,x,h\right)=f_{1}\left(d,x,\pi /4\right)$. Then, one can show that (23) is upper bounded by 1/2.

Subcase 5.2. $x\in [\frac{1}{\sqrt{4+\frac{8}{3}\cos^{2}d}},\frac{1}{\sqrt{4+\frac{8}{3}\sin^{2}d}}]$. One can show that $\max_{g}f_{1}\left(d,x,g\right)$ is achieved when $\sin2g=\frac{3-12x^{2}}{4x^{2}+4x^{2}\cos2d}$ and $\max_{h}f_{2}\left(d,x,h\right)=f_{1}\left(d,x,\pi /4\right)$. Then, one can show that (23) is upper bounded by 3/8.

Subcase 5.3. $x\in [\frac{1}{\sqrt{4+\frac{8}{3}\sin^{2}d}},\frac{1}{2}]$. One can show that $\max_{g}f_{1}\left(d,x,g\right)$ is achieved when $\sin2g=\frac{3-12x^{2}}{4x^{2}+4x^{2}\cos2d}$ and $\max_{h}f_{2}\left(d,x,h\right)$ is achieved when $\sin2h=\frac{3-12x^{2}}{4x^{2}-4x^{2}\cos2d}$. Then, one can show that (23) is upper bounded by 3/8.

We have shown that (23) is upper bounded by 1/2, i.e., λ+μ≤1/2.

This completes the proof. ☐

### 3.2. Conjecture 1 with Non-Normal Matrices *X*: Family 2

**Definition** **3.** *Let $P_{2}$ be the subset of matrices X with d=4, such that A is normal, i.e., $A∼\left[\begin{array}{cccc} a_{1} & 0 & 0 & 0\\ 0 & a_{2} & 0 & 0\\ 0 & 0 & a_{3} & 0\\ 0 & 0 & 0 & a_{4} \end{array}\right]$, and $B∼\left[\begin{array}{cccc} 0 & b_{1} & 0 & 0\\ 0 & 0 & b_{2} & 0\\ 0 & 0 & 0 & b_{1}e^{i\theta_{1}}\\ b_{2}e^{i\theta_{2}} & 0 & 0 & 0 \end{array}\right]$ or A and B switch from each other. The following constraints should be satisfied.*

(26) $$\sum_{i=1}^{4}a_{i}=0,\;\sum_{i=1}^{4}|a_{i}{|}^{2}=\frac{1}{4}-2(|b_{1}{|}^{2}+|b_{2}{|}^{2}).$$

We present the main result of this subsection in Theorem 2. Lemma 8 will be used in the proof of Theorem 2.

**Lemma** **8.** *Suppose $x_{1},\cdots ,x_{n}$ are n complex variables and c is a complex constant. If the $x_{i}$’s satisfy the constraint:*

(27) $$\sum_{i=1}^{n}x_{i}=c,\;\forall c\in C,$$

*the following equality holds by setting $x_{i}=\frac{c}{n}$.*

(28) $$\min(\sum_{i=1}^{n}|x_{i}{|}^{2})=\frac{1}{n}{\left|c\right|}^{2}.$$

**Theorem** **2.** *The inequality (3) holds when $X\in P_{2}$.*

**Proof** **of** **Theorem** **2.** For Lemma 1 (iii) and (iv), we assume $A=\left[\begin{array}{cccc} a_{1} & 0 & 0 & 0\\ 0 & a_{2} & 0 & 0\\ 0 & 0 & a_{3} & 0\\ 0 & 0 & 0 & a_{4} \end{array}\right]$ and $B=\left[\begin{array}{cccc} 0 & b_{1} & 0 & 0\\ 0 & 0 & b_{2} & 0\\ 0 & 0 & 0 & b_{1}e^{i\theta_{1}}\\ b_{2}e^{i\theta_{2}} & 0 & 0 & 0 \end{array}\right]$. Applying a local unitary on *X*, we may assume that $b_{1},b_{2}\geq 0$ and $\theta_{1}=0$. Then, one can show:

(29) $$\begin{array}{c} X^{†}X={⊕}_{i=1}^{4}M_{i}, \end{array}$$

where:

(30) $$\begin{array}{c} M_{i}=\left[\begin{array}{cccc} |a_{i}{|}^{2}+b_{2}^{2} & b_{1}a_{i}^{*} & 0 & a_{i}b_{2}e^{-i\theta_{2}}\\ a_{i}b_{1} & |a_{i}{|}^{2}+b_{1}^{2} & b_{2}a_{i}^{*} & 0\\ 0 & a_{i}b_{2} & |a_{i}{|}^{2}+b_{2}^{2} & b_{1}a_{i}^{*}\\ b_{2}e^{i\theta_{2}}a_{i}^{*} & 0 & a_{i}b_{1} & |a_{i}{|}^{2}+b_{1}^{2} \end{array}\right]. \end{array}$$

Set:

(31) $$\tilde{M_{i}}=\left[\begin{array}{cccc} |a_{i}{|}^{2}+b_{2}^{2} & b_{1}a_{i}^{*} & 0 & 0\\ a_{i}b_{1} & |a_{i}{|}^{2}+b_{1}^{2} & 0 & 0\\ 0 & 0 & |a_{i}{|}^{2}+b_{2}^{2} & b_{1}a_{i}^{*}\\ 0 & 0 & a_{i}b_{1} & |a_{i}{|}^{2}+b_{1}^{2} \end{array}\right].$$

Then, $\tilde{\lambda_{i}^{1}}$, $\tilde{\lambda_{i}^{2}}$ are the double eigenvalues of $\tilde{M_{i}}$ as follows.

(32) $$\begin{array}{cc} {\tilde{\lambda }}_{i}^{1} & =\frac{2|a_{i}{|}^{2}+b_{1}^{2}+b_{2}^{2}+\sqrt{{\left(b_{1}^{2}-b_{2}^{2}\right)}^{2}+4{\left|a_{i}\right|}^{2}b_{1}^{2}}}{2},\\ {\tilde{\lambda }}_{i}^{2} & =\frac{2|a_{i}{|}^{2}+b_{1}^{2}+b_{2}^{2}-\sqrt{{\left(b_{1}^{2}-b_{2}^{2}\right)}^{2}+4{\left|a_{i}\right|}^{2}b_{1}^{2}}}{2}. \end{array}$$

One can show the characteristic polynomial of $M_{i}$ as follows.

(33) $$\begin{array}{cc} \det(\lambda I-M_{i}) & ={\left(\left(\lambda -{\tilde{\lambda }}_{i}^{1}\right)\left(\lambda -{\tilde{\lambda }}_{i}^{2}\right)\right)}^{2}-p\left(\lambda^{2}-\left({\tilde{\lambda }}_{i}^{1}+{\tilde{\lambda }}_{i}^{2}\right)\lambda +r\right), \end{array}$$

where ${\tilde{\lambda }}_{i}^{1},{\tilde{\lambda }}_{i}^{2}$ are given in Equation (32) and:

(34) $$\begin{array}{cc} p & =2|a_{i}{|}^{2}b_{2}^{2},\\ r & =\frac{1}{p}\left(2|a_{i}{|}^{2}b_{1}^{2}b_{2}^{4}+2|a_{i}{|}^{6}b_{2}^{2}+2|a_{i}{|}^{4}b_{1}^{2}b_{2}^{2}+{\left|a_{i}\right|}^{4}b_{2}^{4}+a_{i}^{4}{\left(b_{1}^{2}b_{2}^{2}e^{i\theta_{2}}\right)}^{*}+{\left(a_{i}^{4}\right)}^{*}b_{1}^{2}b_{2}^{2}e^{i\theta_{2}}\right). \end{array}$$

We have the four roots of the equation $\det\lambda (I-M_{i})=0$ as follows.

(35) $$\begin{array}{cc} \lambda_{i}^{1} & =\frac{1}{2}\left({\tilde{\lambda }}_{i}^{1}+{\tilde{\lambda }}_{i}^{2}+\sqrt{2p+{\left({\tilde{\lambda }}_{i}^{1}-{\tilde{\lambda }}_{i}^{2}\right)}^{2}+2\sqrt{p^{2}+4pr-4{\tilde{\lambda }}_{i}^{1}{\tilde{\lambda }}_{i}^{2}}}\right),\\ \lambda_{i}^{2} & =\frac{1}{2}\left({\tilde{\lambda }}_{i}^{1}+{\tilde{\lambda }}_{i}^{2}+\sqrt{2p+{\left({\tilde{\lambda }}_{i}^{1}-{\tilde{\lambda }}_{i}^{2}\right)}^{2}-2\sqrt{p^{2}+4pr-4{\tilde{\lambda }}_{i}^{1}{\tilde{\lambda }}_{i}^{2}}}\right),\\ \lambda_{i}^{3} & =\frac{1}{2}\left({\tilde{\lambda }}_{i}^{1}+{\tilde{\lambda }}_{i}^{2}-\sqrt{2p+{\left({\tilde{\lambda }}_{i}^{1}-{\tilde{\lambda }}_{i}^{2}\right)}^{2}-2\sqrt{p^{2}+4pr-4{\tilde{\lambda }}_{i}^{1}{\tilde{\lambda }}_{i}^{2}}}\right),\\ \lambda_{i}^{4} & =\frac{1}{2}\left({\tilde{\lambda }}_{i}^{1}+{\tilde{\lambda }}_{i}^{2}-\sqrt{2p+{\left({\tilde{\lambda }}_{i}^{1}-{\tilde{\lambda }}_{i}^{2}\right)}^{2}+2\sqrt{p^{2}+4pr-4{\tilde{\lambda }}_{i}^{1}{\tilde{\lambda }}_{i}^{2}}}\right). \end{array}$$

Substituting Equation (34) into Equation (35), we have the sum of the largest two eigenvalues, which belong to exact one $M_{i}$ as follows.

(36) $$\begin{array}{c} \lambda_{i}^{1}+\lambda_{i}^{2}\\ =2|a_{i}{|}^{2}+b_{1}^{2}+b_{2}^{2}\\ +\frac{1}{2}\sqrt{{\left(b_{1}^{2}-b_{2}^{2}\right)}^{2}+4{\left|a_{i}b_{1}+a_{i}^{*}b_{2}e^{i\frac{\theta_{2}}{2}}\right|}^{2}}\\ +\frac{1}{2}\sqrt{{\left(b_{1}^{2}-b_{2}^{2}\right)}^{2}-4{\left|a_{i}b_{1}+a_{i}^{*}b_{2}e^{i\frac{\theta_{2}}{2}}\right|}^{2}}. \end{array}$$

We also have the sum of the largest two eigenvalues from two different submatrices $M_{i},M_{j}$ as follows.

(37) $$\begin{array}{c} \lambda_{i}^{1}+\lambda_{j}^{2}\\ =|a_{i}{|}^{2}+{\left|a_{j}\right|}^{2}+b_{1}^{2}+b_{2}^{2}\\ +\frac{1}{2}\sqrt{{\left(b_{1}^{2}-b_{2}^{2}\right)}^{2}+4{\left|a_{i}b_{1}+a_{i}^{*}b_{2}e^{i\frac{\theta_{2}}{2}}\right|}^{2}}\\ +\frac{1}{2}\sqrt{{\left(b_{1}^{2}-b_{2}^{2}\right)}^{2}+4{\left|a_{j}b_{1}+a_{j}^{*}b_{2}e^{i\frac{\theta_{2}}{2}}\right|}^{2}}. \end{array}$$

Next, we need to show that both Equations (36) and (37) are upper bounded by $\frac{1}{2}$.

We firstly consider Equation (36). Suppose $b_{1}^{2}+b_{2}^{2}=k$. It follows from Equation (26) that $\sum_{i=1}^{4}a_{i}=0$ and $\sum_{i=1}^{4}{\left|a_{i}\right|}^{2}=\frac{1}{4}-2k$. It is safe to suppose $|a_{1}|$ is the maximum. Then, we have $\sum_{i=2}^{4}a_{i}=-a_{1}$. View $a_{2},a_{3},a_{4}$ as the three variables $x_{1},x_{2},x_{3}$ in Lemma 8 and $-a_{1}$ as the constant *c* in Lemma 8. Then, Lemma 8 implies $\min\sum_{i=2}^{4}|a_{i}{|}^{2}=\frac{1}{3}{\left|a_{1}\right|}^{2}$ by setting $a_{2}=a_{3}=a_{4}=-\frac{a_{1}}{3}$. From $\sum_{i=1}^{4}{\left|a_{i}\right|}^{2}=\frac{1}{4}-2k$, we obtain $\frac{4}{3}{\left|a_{1}\right|}^{2}=\frac{1}{4}-2k$. Therefore, we have $\max|a_{i}{|}^{2}=\frac{3}{4}\left(\frac{1}{4}-2k\right)$. Set $b_{1}^{2}=x,b_{2}^{2}=k-x,a_{i}=\sqrt{\frac{3}{4}\left(\frac{1}{4}-2k\right)}e^{ii}$. Then, Equation (36) can be transformed into the following function.

(38) $$\begin{array}{cc} f(x) & =\frac{3}{8}-2k\\ & +\frac{1}{2}\sqrt{{\left(2x-k\right)}^{2}+3\left(\frac{1}{4}-2k\right)\left(2\sqrt{x(k-x)}\cos\beta +k\right)}\\ & +\frac{1}{2}\sqrt{{\left(2x-k\right)}^{2}+3\left(\frac{1}{4}-2k\right)\left(-2\sqrt{x(k-x)}\cos\beta +k\right)},\;\beta =2\alpha_{i}-\frac{\theta_{2}}{2}. \end{array}$$

Then, we have the derivative of *f* as follows.

(39) $$\begin{array}{cc} \frac{\partial f}{\partial x} & =\frac{f_{1}\left(x\right)}{f_{2}\left(x\right)}, \end{array}$$

where:

(40) $$\begin{array}{cc} f_{1}\left(x\right) & =2\left(k-2x\right)|a_{i}{|}^{2}\cos\beta \left(t_{1}-t_{2}\right)-2\left(k-2x\right)\sqrt{(k-x)x}\left(t_{1}+t_{2}\right),\\ f_{2}\left(x\right) & =\sqrt{(k-x)x}t_{1}t_{2},\\ t_{1} & =\sqrt{{\left(k-2x\right)}^{2}+4k|a_{i}{|}^{2}-8{\left|a_{i}\right|}^{2}\cos\beta \sqrt{(k-x)x}}\\ & -\sqrt{{\left(k-2x\right)}^{2}+4k|a_{i}{|}^{2}+8{\left|a_{i}\right|}^{2}\cos\beta \sqrt{(k-x)x}},\\ t_{2} & =\sqrt{{\left(k-2x\right)}^{2}+4k|a_{i}{|}^{2}-8{\left|a_{i}\right|}^{2}\cos\beta \sqrt{(k-x)x}}\\ & +\sqrt{{\left(k-2x\right)}^{2}+4k|a_{i}{|}^{2}+8{\left|a_{i}\right|}^{2}\cos\beta \sqrt{(k-x)x}}. \end{array}$$

One can show that $x=0,\frac{k}{2},k$ are three extreme points of f(x). We have $f\left(0\right)=f\left(k\right)=\frac{3}{8}-2k+\sqrt{\frac{3k}{4}-5k^{2}}$. The maximum of f(0) is $\frac{9}{20}$ when $x=\frac{1}{40}$. We have $f\left(\frac{k}{2}\right)=\frac{3}{8}-2k+\sqrt{\frac{3}{4}\left(\frac{1}{4}-2k\right)\left(k+k\cos\beta \right)}+\sqrt{\frac{3}{4}\left(\frac{1}{4}-2k\right)\left(k-k\cos\beta \right)}$. The maximum of $f(\frac{k}{2})$ is $\frac{4+\sqrt{10}}{16}\left(<\frac{9}{20}\right)$ when $\cos\beta =0,k=\frac{5-\sqrt{10}}{80}$. Therefore, Equation (36) is upper bounded by $\frac{1}{2}$.

We next consider Equation (37) and suppose $b_{1}^{2}+b_{2}^{2}=k,b_{1}^{2}=x,b_{2}^{2}=k-x,a_{i}=|a_{i}{|}^{2}e^{i\alpha_{i}},a_{j}={\left|a_{j}\right|}^{2}e^{i\alpha_{j}}$ in the same way. One can show that the maximum of Equation (37) with $|a_{i}{|}^{2}+{\left|a_{j}\right|}^{2}=c$ can be reached when $|a_{i}\left|=\right|a_{j}|$ and $\cos\left(2\alpha_{i}-\frac{\theta_{2}}{2}\right)=\cos\left(2\alpha_{j}-\frac{\theta_{2}}{2}\right)=0$. Therefore, in order to maximize (37), we can set $|a_{i}{|}^{2}={\left|a_{j}\right|}^{2}=\frac{1}{8}-k$. Then, Equation (37) can be transformed into the following function.

(41) $$\begin{array}{cc} g(x) & =\frac{1}{4}-k+\sqrt{{\left(2x-k\right)}^{2}+\left(\frac{1}{2}-4k\right)\left(k+2\sqrt{x(k-x)}\right)}. \end{array}$$

One can show that the maximum of g(x) can only appear when $x=0,k,\frac{4k-\sqrt{-1+16k-48k^{2}}}{8},\frac{4k+\sqrt{-1+16k-48k^{2}}}{8}$. Then, we have $g\left(0\right)=g\left(k\right)=\frac{1}{4}-k+\sqrt{\frac{k}{2}-3k^{2}}$ and $g\left(\frac{4k-\sqrt{-1+16k-48k^{2}}}{8}\right)=g\left(\frac{4k+\sqrt{-1+16k-48k^{2}}}{8}\right)=\frac{1}{4}\sqrt{16k^{2}-8k+1}$. One can show that both $\frac{1}{4}-k+\sqrt{\frac{k}{2}-3k^{2}}$ and $\frac{1}{4}\sqrt{16k^{2}-8k+1}$ are upper bounded by $\frac{1}{2}$ when $k\in [0,\frac{1}{8}]$.

Therefore, we conclude that the sum of the largest two eigenvalues of $X^{†}X$ in Equation (29) is upper bounded by $\frac{1}{2}$. This completes the proof. ☐

### 3.3. Conjecture 1 with Non-Normal Matrices *X*: Family 3

In this subsection, we investigate Conjecture 1 with *X* for d≥5 defined as follows.

**Definition** **4.** *Let $P_{3}$ be the subset of matrices X with d≥5, such that $A∼D_{A}P_{A},B∼D_{B}P_{B}$, where $D_{A}=\mathrm{diag}(a_{1},a_{2},\cdots ,a_{d})$, $D_{B}=\mathrm{diag}(b_{1},b_{2},\cdots ,b_{d})$, and $P_{A},P_{B}$ are permutation matrices with zero-diagonals. The parameters $a_{i}$’s, $b_{i}$’s satisfy the following constraint:*

(42) $$\begin{array}{c} \sum_{i=1}^{d}\left(\right|a_{i}{|}^{2}+|b_{i}{|}^{2})=\frac{1}{d}. \end{array}$$

It is not an easy task to characterize the characteristic polynomial of $X^{†}X$, especially when the dimension is large. In Theorem 3, we use the Gershgorin circle theorem and Brauer theorem to study Conjecture 1. They are two important theorems in the field of the localization of eigenvalues. The following fact will be used in the proof of Theorem 3.

It follows from (1) that $X^{†}X=H_{1}+H_{2}$ where:

(43) $$\begin{array}{ccc} H_{1} & := & A^{†}A⊗I+I⊗B^{†}B,\\ H_{2} & := & A^{†}⊗B+A⊗B^{†}. \end{array}$$

Furthermore, the first equation of (2) implies that $\mathrm{Tr}H_{2}=0$, and the second equation of (2) implies that $\mathrm{Tr}X^{†}X=\mathrm{Tr}H_{1}=\sum_{j=1}^{d^{2}}\sigma_{j}^{2}=1$. Therefore, $X^{†}X$ can be regarded as a normalized quantum state in terms of quantum physics.

**Theorem** **3.** *The inequality (3) holds for d≥5 when $X\in P_{3}$.*

**Proof** **of** **Theorem** **3.** Recall that $H_{1}$ in Equation (43) is diagonal and $H_{2}$ in Equation (43) is a Hermitian matrix with zero-diagonal. There exist two permutations σ and τ with σ(k)≠k,τ(k)≠k,∀k∈{1,⋯,d}, which are respectively equivalent to $P_{A}$ and $P_{B}$. We find that the (k,σ(k)) entry of *A* is $a_{k}$ and the (k,τ(k)) entry of *B* is $b_{k}$. Therefore, the (σ(k),k) entry of *A*^†^ is $a_{k}^{*}$, and the (τ(k),k) entry of *B*^†^ is $b_{k}^{*}$. They imply that there are exactly two entries in each row of $H_{2}$, which can be expressed with $a_{i},b_{j}$ and their conjugates, for i,j∈{1,⋯,d}. This implies that the two elements $a_{\sigma^{-1}\left(i\right)}^{*}b_{j}$, which is the $\left(d\left(i-1\right)+j,d(\sigma^{-1}\left(i\right)-1)+\tau \left(j\right)\right)$ entry of $X^{†}X$, and $a_{i}b_{\tau^{-1}\left(j\right)}^{*}$, which is the $\left(d\left(i-1\right)+j,d\left(\sigma \left(i\right)-1\right)+\tau^{-1}\left(j\right)\right)$ entry of $X^{†}X$, are in the same the (d(i−1)+j)-th row of $X^{†}X$ and that both are non-diagonal entries of $X^{†}X$. Further, the diagonal entry of $X^{†}X$ in this row is $\left(|a_{\sigma^{-1}\left(i\right)}{|}^{2}+|b_{\tau^{-1}\left(j\right)}{|}^{2}\right)$. Recall that σ(i)≠i,τ(i)≠i,∀i∈{1,⋯,d}. Equation (5) implies that the largest eigenvalue $\lambda_{1}$ of $X^{†}X$ satisfies:

(44) $$\begin{array}{ccc} \lambda_{1} & \leq & \max_{i,j}\left(|a_{\sigma^{-1}\left(i\right)}{|}^{2}+|b_{\tau^{-1}\left(j\right)}{|}^{2}+|a_{\sigma^{-1}\left(i\right)}\left|\right|b_{j}\left|+\right|a_{i}\left|\right|b_{\tau^{-1}\left(j\right)}|\right). \end{array}$$

Applying the basic inequality, we obtain $|a_{p}\left|\right|b_{q}\left|+\right|a_{s}\left|\right|b_{t}\left|\leq (\right|a_{p}{|}^{2}+|b_{q}{|}^{2}+|a_{s}{|}^{2}+|b_{t}{|}^{2})/2$. Since p≠s and q≠t, the constraint (42) implies that $|a_{i}{|}^{2}+{\left|b_{j}\right|}^{2}\leq \frac{1}{d}$ and $|a_{p}\left|\right|b_{q}\left|+\right|a_{s}\left|\right|b_{t}|\leq \frac{1}{2d}$. Hence, we have $\lambda_{1}\leq \frac{3}{2d}$, and thus, $\lambda_{1}+\lambda_{2}\leq \frac{3}{d}$. This implies Conjecture 1 holds for d≥6. Next, we will prove that Conjecture 1 holds for d=5.

Let us recall (43). The inequality (7) implies that the second largest eigenvalue of $X^{†}X$ satisfies $\lambda_{2}\left(X^{†}X\right)\leq \lambda_{2}\left(H_{1}\right)+\lambda_{1}\left(H_{2}\right)$, where $\lambda_{2}\left(H_{1}\right)$ means the second largest eigenvalue of $H_{1}$ and $\lambda_{1}\left(H_{2}\right)$ means the largest eigenvalue of $H_{2}$. Since $H_{1}$ is diagonal, $\lambda_{2}\left(H_{1}\right)$ is the second largest diagonal element of $H_{1}$. Applying Lemma 4 to $H_{2}$, one can show that $\lambda_{1}\left(H_{2}\right)$ should not be greater than the largest root of these quadratic polynomials λ2=∑k=1k≠k1n|X†X(k1,k)|∑k=1k≠k2n|X†X(k2,k)| for $k_{1}\neq k_{2}$. Suppose $k_{1}=d\left(i-1\right)+j$ and $k_{2}=d\left(p-1\right)+q$ with (i,j)≠(p,q). Hence, we can bound $\lambda_{1}\left(X^{†}X\right)+\lambda_{2}\left(X^{†}X\right)$ as follows.

(45) $$\begin{array}{c} \left(\lambda_{1}+\lambda_{2}\right)\leq \max_{\left(i,j)\neq (p,q\right)}\left(|a_{\sigma^{-1}\left(i\right)}{|}^{2}+|b_{\tau^{-1}\left(j\right)}{|}^{2}+|a_{\sigma^{-1}\left(p\right)}{|}^{2}+{\left|b_{\tau^{-1}\left(q\right)}\right|}^{2}+2\sqrt{c}\right), \end{array}$$

where $c=\left(|a_{\sigma^{-1}\left(i\right)}\left|\right|b_{j}\left|+\right|a_{i}\left|\right|b_{\tau^{-1}\left(j\right)}|\right)\left(|a_{\sigma^{-1}\left(p\right)}\left|\right|b_{q}\left|+\right|a_{p}\left|\right|b_{\tau^{-1}\left(q\right)}|\right)$.

Suppose $|a_{i}|$ and $|b_{j}|$ are in the decreasing order. In order to maximize the rhs of (45), we can assume that $x_{1}=\left|a_{1}\right|$, $x_{2}=\left|a_{2}\right|$, $x_{3}=\left|b_{1}\right|$ and $x_{4}=\left|b_{2}\right|$ and other $|a_{i}|$’s and $|b_{j}|$’s are zero. Then, our problem can be transformed into an optimization task as follows.

(46) $$\begin{array}{cc} \max & \;f\left(x_{1},x_{2},x_{3},x_{4}\right)=2x_{1}^{2}+x_{3}^{2}+x_{4}^{2}+2\sqrt{\left(x_{1}x_{3}+x_{2}x_{4}\right)\left(x_{1}x_{4}+x_{2}x_{3}\right)}\\ s.t. & \;x_{i}>0,\;i=1,2,3,4,\\ & \;\sum_{i=1}^{4}x_{i}^{2}=\frac{1}{d},\\ & x_{1}-x_{2}\geq 0,\;x_{3}-x_{4}\geq 0,\\ & x_{1}^{2}-x_{2}^{2}\geq x_{3}^{2}-x_{4}^{2}. \end{array}$$

Since $\sum_{i=1}^{4}x_{i}^{2}=\frac{1}{d}$, we let $x_{1}=\sqrt{\frac{1}{d}}\left(\cos a\cos b\right)$, $x_{2}=\sqrt{\frac{1}{d}}\left(\cos a\sin b\right)$, $x_{3}=\sqrt{\frac{1}{d}}\left(\sin a\cos c\right)$ and $x_{4}=\sqrt{\frac{1}{d}}\left(\sin a\sin c\right)$. Then, we obtain $f=\frac{1}{4d}\left(4+\cos2\left(a-b\right)+2\cos2b+\cos2\left(a+b\right)+2\sqrt{2}\sqrt{\sin^{2}2a\left(\sin2b+\sin2c\right)}\right)$, which implies *f* obtains its maximum only when sin2c=1. Furthermore, sin2c=1 implies $x_{3}=x_{4}$. Substituting $x_{3}=x_{4}$ and $\sum_{i=1}^{4}x_{i}^{2}=\frac{1}{d}$ into $f\left(x_{1},x_{2},x_{3},x_{4}\right)=2x_{1}^{2}+x_{3}^{2}+x_{4}^{2}+2\sqrt{\left(x_{1}x_{3}+x_{2}x_{4}\right)\left(x_{1}x_{4}+x_{2}x_{3}\right)}$, *f* actually is a two-variable function, that is $f\left(x_{1},x_{2}\right)=\frac{1}{d}+x_{1}^{2}-x_{2}^{2}+2\left(x_{1}+x_{2}\right)\sqrt{\frac{1}{2d}-\frac{1}{2}\left(x_{1}^{2}+x_{2}^{2}\right)}$. Let d=5. Based on the optimization task expressed by Equation (46), the following task is specifically to show:

(47) $$\begin{array}{cc} \max & \;f\left(x_{1},x_{2}\right)=\frac{1}{5}+x_{1}^{2}-x_{2}^{2}+2\left(x_{1}+x_{2}\right)\sqrt{\frac{1}{10}-\frac{1}{2}\left(x_{1}^{2}+x_{2}^{2}\right)}\leq \frac{1}{2}\\ s.t. & \;0<x_{2}\leq x_{1},\;x_{1}^{2}+x_{2}^{2}<\frac{1}{5}. \end{array}$$

We first consider the boundary case, i.e., $x_{1}=x_{2}$. In such a case, we have $f_{1}\left(x_{1}\right):=f\left(x_{1},x_{1}\right)=\frac{1}{5}+4x_{1}\sqrt{\frac{1}{10}-x_{1}^{2}}\leq \frac{2}{5}$. This implies that the maximum over the boundary is upper bounded by $\frac{2}{5}$. Then, let us consider the non-boundary case. It is known that the maximum in this case occurs when both partial derivatives of $f(x_{1},x_{2})$ equal zero. By computing, one can show the two partial derivatives of $f(x_{1},x_{2})$ as follows.

(48) $$\begin{array}{cc} \frac{\partial f(x_{1},x_{2})}{\partial x_{1}} & =2x_{1}-\frac{x_{1}\left(x_{1}+x_{2}\right)}{\sqrt{\frac{1}{10}-\frac{x_{1}^{2}+x_{2}^{2}}{2}}}+2\sqrt{\frac{1}{10}-\frac{x_{1}^{2}+x_{2}^{2}}{2}},\\ \frac{\partial f(x_{1},x_{2})}{\partial x_{2}} & =-2x_{2}-\frac{x_{2}\left(x_{1}+x_{2}\right)}{\sqrt{\frac{1}{10}-\frac{x_{1}^{2}+x_{2}^{2}}{2}}}+2\sqrt{\frac{1}{10}-\frac{x_{1}^{2}+x_{2}^{2}}{2}}. \end{array}$$

Set the two partial derivatives in Equation (48) equal to zero. One can solve the root as $x_{1}^{\prime }=\sqrt{\frac{3}{40}+\frac{1}{10\sqrt{2}}},x_{2}^{\prime }=\frac{1}{20}\left(3\sqrt{10(3+2\sqrt{2})}-4\sqrt{5(3+2\sqrt{2})}\right)$. One can further obtain $f(x_{1}^{\prime },x_{2}^{\prime })\approx 0.483$, which is strictly less than $\frac{1}{2}$ by computing. Therefore, combining the above two cases, the inequality in Equation (47) holds.

Till now, all dimensions d≥5 have been studied. This completes the proof. ☐

Combining Lemma 2 and Theorem 3, we have that Conjecture 1 holds when $X\in_{d}\cup_{3}$ with d≥5.

### 3.4. A Sufficient Condition for Conjecture 1

The localization of eigenvalues has been a hot topic of matrix analysis for years. Several results show that eigenvalues can be bounded by traces. In this subsection, we use the recent results on the bounds of eigenvalues to formulate a sufficient condition for Conjecture 1.

**Lemma** **9.** *([22], Theorem 2.1.) Let $A\in H^{n\times n}$ with eigenvalues $\lambda_{1}\geq \lambda_{2}\geq \cdots \geq \lambda_{n}$. Then, for any 1≤k≤n,*

(49) $$\begin{array}{c} |\lambda_{k}-\frac{\mathrm{Tr}A}{n}|\leq \sqrt{\frac{n-1}{n}\left({\left||A|\right|}^{2}-\frac{{\mathrm{Tr}}^{2}A}{n}\right)}. \end{array}$$

*Here, $||A||:={\left(A^{A}\right)}^{1/2}={\left(\sum_{j=1}^{n}\lambda_{j}^{2}\right)}^{1/2}$, i.e., the $l_{2}$-norm.*

Then, we have the following result by using Lemma 9.

**Lemma** **10.** *Conjecture 1 holds for X satisfying ${\left||X^{†}X|\right|}^{2}\leq \frac{1}{10}$ when d=4.*

**Proof** **of** **Lemma** **10.** Let d=4 in Conjecture 1. Since $X^{†}X$ is Hermitian, we have ${\left||X^{†}X|\right|}^{2}=\mathrm{Tr}{\left(X^{†}X\right)}^{2}=\sum_{j=1}^{n}\lambda_{j}^{2}$, where $\lambda_{j}$’s are eigenvalues of $X^{†}X$. Recall that $\mathrm{Tr}X^{†}X=1$. Therefore, ${\left||X^{†}X|\right|}^{2}$ is lower bounded by $\frac{1}{16}$. Applying Lemma 9 to $X^{†}X$, the largest eigenvalue of $X^{†}X$ satisfies:

(50) $$\begin{array}{c} \lambda_{1}\leq \frac{1}{16}+\sqrt{\frac{15}{16}}{\left({\left||X^{†}X|\right|}^{2}-\frac{1}{16}\right)}^{1/2}. \end{array}$$

One can show that ${\left||X^{†}X|\right|}^{2}\leq \frac{1}{10}$ is equivalent to $\frac{1}{16}+\sqrt{\frac{15}{16}}{\left({\left||X^{†}X|\right|}^{2}-\frac{1}{16}\right)}^{1/2}\leq \frac{1}{4}$. This implies that ${\left||X^{†}X|\right|}^{2}\leq \frac{1}{10}$ suffices to obtain $\lambda_{1}+\lambda_{2}\leq \frac{1}{2}$. This completes the proof. ☐

## 4. Discussion

It is known that all entangled two-qubit states are distillable; but for 3⊗3 or 2⊗4 systems, there exist bound entangled states: entangled states that cannot be distilled [9]. Furthermore, 2×2 PPT (positive partial transpose) states, which are defined in Section 1, are separable, while 3×3 PPT states may be entangled. This indicates that 3×3 non-distillable states exist. View the two sets of 2×2 PPT states and 3×3 PPT states as two balls, respectively. One can find the difference between the two balls geometrically.

Quantum communications need pure entangled states, which can be distilled from not completely entangled states. The distillability of bipartite states is connected with the possibility of obtaining asymptotically pure maximally-entangled states by LOCC from many copies of a given state. Such maximally-entangled states can be then used for transmitting qubits by means of teleportation. This is why entanglement distillation plays a fundamental role in quantum information.

Definition 1 shows that if Conjecture 1 with d=4 were true, it is still possible that Werner states might be *n*-distillable with some integer n>2. However it is widely believed that Werner states with α=−1/2 may be undistillable [1,2,9,23,24].

To sum up, in this paper, we introduced a main open problem in quantum information, i.e., the distillability problem. One way to attack the problem is to solve the equivalent mathematical problem Conjecture 1. It is known that Conjecture 1 holds for the set of normal matrices with d≥4. We carried out the first step to prove Conjecture 1 for non-normal matrices and, thus, the distillability problem.

## Appendix A. The Brief Proof of Lemma 2 and Some Related New Findings

**Proof** **of** **Lemma** **2.** It is easy to verify that the operator *X* in (1) is normal iff operators *A* and *B* are normal. Since *X* is normal, we can replace singular values with moduli of eigenvalues, which means:

(A1) $$\begin{array}{c} \lambda_{ij}=a_{i}+b_{j} \end{array}$$

where $a_{i}$ and $b_{j}$ are eigenvalues of *A* and *B*, respectively, and $\lambda_{ij}$ are eigenvalues of *X*. We then have:

(A2) supX∈Nd(σ12+σ22)=supX∈Nd(|λ1|2+|λ2|2)=supX∈Ndmaxi,j,k,l∈{1,⋯,d},(i,j)≠(k,l)(|ai+bj|2+|ak+bl|2),

where $\lambda_{1}$ and $\lambda_{2}$ are two eigenvalues of *X* with the largest moduli. From (2), parameters $a_{i}$’s and $b_{j}$’s should satisfy:

(A3) $$\begin{array}{c} \sum_{i=1}^{d}a_{i}=\mathrm{Tr}A=0,\;\sum_{j=1}^{d}b_{j}=\mathrm{Tr}B=0, \end{array}$$

(A4) $$\begin{array}{c} \sum_{i=1}^{d}|a_{i}{|}^{2}+\sum_{j=1}^{d}{\left|b_{j}\right|}^{2}=\mathrm{Tr}A^{†}A+\mathrm{Tr}B^{†}B=\frac{1}{d}. \end{array}$$

The proof of [9] (Theorem 1) shows that (A2) is further equivalent to the following term.

(A5) $$\begin{array}{cc} \underset{X\in N_{d}}{\sup}\left(\sigma_{1}^{2}+\sigma_{2}^{2}\right) & =\underset{X\in N_{d}}{\sup}\max\{|a_{1}+b_{1}{|}^{2}+|a_{2}+b_{2}{|}^{2},\;|a_{1}+b_{1}{|}^{2}+|a_{1}+b_{2}{|}^{2}\}. \end{array}$$

Then, we have that the following two inequalities hold:

(A6) $$\begin{array}{cc} |a_{1}+b_{1}{|}^{2}+{\left|a_{2}+b_{2}\right|}^{2} & \leq \frac{1}{2}, \end{array}$$

(A7) $$\begin{array}{cc} |a_{1}+b_{1}{|}^{2}+{\left|a_{1}+b_{2}\right|}^{2} & \leq \frac{1}{2}, \end{array}$$

under the constraints (A3) and (A4). The first inequality follows from the identity:

(A8) $$\begin{array}{c} {\left|x+y\right|}^{2}={2(|x|}^{2}+{\left|y\right|}^{2}{)-|x-y|}^{2}\leq {2(|x|}^{2}+{\left|y\right|}^{2}) \end{array}$$

which implies:

(A9) $$\begin{array}{cc} |a_{1}+b_{1}{|}^{2}+{\left|a_{2}+b_{2}\right|}^{2} & \leq 2(|a_{1}{|}^{2}+|b_{1}{|}^{2}+|a_{2}{|}^{2}+|b_{2}{|}^{2})\\ & \leq \frac{2}{d}=\frac{1}{2}. \end{array}$$

The second inequality follows from the following Proposition A1 proven by [9] (Proposition 6). This completes the proof. ☐

**Proposition** **A1.**
*Suppose $\vec{a}$ and $\vec{b}$ are d≥3-dimensional vectors with complex elements $\tilde{a_{i}}$ and $\tilde{b_{i}}$ satisfying the constraints:*

(A10) $$\begin{array}{c} \sum_{i=1}^{d}\tilde{a_{i}}=\sum_{i=1}^{d}\tilde{b_{i}}=0,\;\;\;\;\;\;\sum_{i=1}^{d}|\tilde{a_{i}}{|}^{2}+\sum_{i=1}^{d}{\left|\tilde{b_{i}}\right|}^{2}=\frac{1}{d}. \end{array}$$

*Then, the following equality:*

(A11) $$\begin{array}{c} \max_{\vec{a},\vec{b}}\left(\right|\tilde{a_{1}}+\tilde{b_{1}}{|}^{2}+|\tilde{a_{1}}+\tilde{b_{2}}{|}^{2})=\frac{3d-4}{d^{2}} \end{array}$$

*holds.*

Following the ideas in the proof of [9] (Proposition 6), one can similarly obtain the corollary as follows.

**Corollary** **A1.**
*Suppose $k\in [0,\frac{1}{d}]$, and $\vec{a}$ and $\vec{b}$ are d≥3-dimensional vectors with complex elements $\tilde{a_{i}}$ and $\tilde{b_{i}}$ satisfying the constraints:*

(A12) $$\begin{array}{c} \sum_{i=1}^{d}\tilde{a_{i}}=\sum_{i=1}^{d}\tilde{b_{i}}=0,\;\;\;\;\;\;\sum_{i=1}^{d}|\tilde{a_{i}}{|}^{2}+\sum_{i=1}^{d}{\left|\tilde{b_{i}}\right|}^{2}=\frac{1}{d}-k. \end{array}$$

*Then, the following equality:*

(A13) $$\begin{array}{c} \max_{\vec{a},\vec{b}}\left(\right|\tilde{a_{1}}+\tilde{b_{1}}{|}^{2}+|\tilde{a_{1}}+\tilde{b_{2}}{|}^{2})=\frac{\left(3d-4)(1-dk\right)}{d^{2}} \end{array}$$

*holds.*

The maximum can be reached when:

(A14) $$\begin{array}{cccc} \tilde{a_{1}} & =\frac{d-1}{d}\sqrt{\frac{2(1-dk)}{3d-4}},\; & \tilde{a_{i}}=-\frac{1}{d}\sqrt{\frac{2(1-dk)}{3d-4}}, & (i>1),\\ \tilde{b_{1}} & =\tilde{b_{2}}=\frac{d-2}{d}\sqrt{\frac{1-dk}{2(3d-4)}},\; & \tilde{b_{i}}=-\frac{2}{d}\sqrt{\frac{1-dk}{2(3d-4)}}, & (i>2). \end{array}$$

Next, we show that Conjecture 1 no longer holds when d=3. Therefore, we claim that d≥4 is essential to Conjecture 1.

**Lemma** **A1.**
*Let $N_{d}$ be a subset of normal operators X in (1) satisfying the constraints (2). Then, for d=3, we have:*

(A15) $$\begin{array}{c} \frac{5}{9}\leq \underset{X\in N_{d}}{\sup}\left(\sigma_{1}^{2}+\sigma_{2}^{2}\right)\leq \frac{2}{3}, \end{array}$$

*where $\sigma_{1}$ and $\sigma_{2}$ are the two largest singular values of operator X.*

**Proof** **of** **Lemma** **A1.** According to (A8), we have:

(A16) $$\begin{array}{cc} |a_{1}+b_{1}{|}^{2}+{\left|a_{2}+b_{2}\right|}^{2} & \leq 2(|a_{1}{|}^{2}+|b_{1}{|}^{2}+|a_{2}{|}^{2}+|b_{2}{|}^{2})\leq \frac{2}{d}=\frac{2}{3}. \end{array}$$

Proposition A1 implies that:

(A17) $$\begin{array}{c} \max(|a_{1}+b_{1}{|}^{2}+|a_{1}+b_{2}{|}^{2})=\frac{3d-4}{d^{2}}=\frac{5}{9}. \end{array}$$

Due to the relation (A5), we obtain (A15) for d=3. ☐

## References

[1] DiVincenzo D.P., Shor P.W., Smolin J.A., Terhal B.M., Thapliyal A.V. (2000). Evidence for bound entangled states with negative partial transpose. Phys. Rev. A.

[2] Dür W., Cirac J.I., Lewenstein M., Bruß D. (2000). Distillability and partial transposition in bipartite systems. Phys. Rev. A.

[3] Terhal B.M. (2000). A family of indecomposable positive linear maps based on entangled quantum states. Linear Algebra Its Appl..

[4] Poon E. (2015). Preservers of maximally entangled states. Linear Algebra Its Appl..

[5] Cariello D. (2016). A gap for PPT entanglement. Linear Algebra Its Appl..

[6] Hou J., Qi X. (2013). Linear maps preserving separability of pure states. Linear Algebra Its Appl..

[7] Alfsen E., Shultz F. (2012). Finding decompositions of a class of separable states. Linear Algebra Its Appl..

[8] Woerdeman H.J. (2004). The separability problem and normal completions. Linear Algebra Its Appl..

[9] Pankowski L., Piani M., Horodecki M., Horodecki P. (2010). A few steps more towards npt bound entanglement. IEEE. Trans. Inf. Theory.

[10] Vianna R.O., Doherty A.C. (2006). Distillability of Werner states using entanglement witnesses and robust semidefinite programs. Phys. Rev. A.

[11] Bandyopadhyay S., Roychowdhury V. (2003). Classes of *n*-copy undistillable quantum states with negative partial transposition. Phys. Rev. A.

[12] Chen L., Chen Y.-X. (2008). Rank-three bipartite entangled states are distillable. Phys. Rev. A.

[13] Horodecki M., Horodecki P., Horodecki R. (1997). Inseparable two spin- $\frac{1}{2}$ density matrices can be distilled to a singlet form. Phys. Rev. Lett. Phys. Rev. A.

[14] Rains E.M. (1999). Bound on distillable entanglement. Phys. Rev. A.

[15] Chen L., Dokovic D.Z. (2011). Distillability and PPT entanglement of low-rank quantum states. J. Phys. A.

[16] Chen L., Dokovic D.Z. (2016). Distillability of non-positive-partial-transpose bipartite quantum states of rank four. Phys. Rev. A.

[17] Peres A. (1996). Separability criterion for density matrices. Phys. Rev. Lett..

[18] Horodecki M., Horodecki P., Horodecki R. (1996). Separability of mixed states: necessary and sufficient conditions. Phys. Lett. A.

[19] Werner R.F. (1989). Quantum states with Einstein-Podolsky-Rosen correlations admitting a hidden-variable model. Phys. Rev. A.

[20] Bernstein D. (2009). Matrix Mathematics: Theory, Facts, and Formulas.

[21] Horn R., Johnson C. (1985). Matrix Analysis.

[22] Huang T., Wang L. (2007). Improving eigenvalues of complex matrices using traces. Linear Algebra Its Appl..

[23] Hiroshima T. (2008). Bound entangled states with non-positive partial transpose exist. arXiv.

[24] Hiroshima T. (2012). A problem of existence of bound entangled states with non-positive partial transpose and the hilbert’s 17th problem. arXiv.

